# Case Report: Neuropsychological Findings in IgLON5 Antibody Disorder

**DOI:** 10.3389/fneur.2021.632497

**Published:** 2021-02-03

**Authors:** Jasmin El Shazly, Martin Juenemann, Tibo Gerriets, Marlene Tschernatsch

**Affiliations:** ^1^Department of Neurology, Heart and Brain Research Group, University Hospital Giessen and Marburg, Giessen, Germany; ^2^Department of Psychocardiology, Kerckhoff Klinik, Bad Nauheim, Germany; ^3^Department of Neurology, Justus Liebig University, Giessen, Germany; ^4^Department of Neurology, Gesundheitszentrum Wetterau, Bad Nauheim, Germany

**Keywords:** IgLON5, sleep disorder, cognitive deficits, neuropsychological findings, autoimmune encephalopathy

## Abstract

IgLON5 antibody encephalopathy is a rare but increasingly recognized disorder with a variety of clinical signs. Typical symptoms are sleep disorder, gait disturbances, signs of bulbar dysfunction and a variety of neurological symptoms like oculomotor abnormalities and movement disorders. In addition, cognitive decline can be a prominent symptom. So far, there are only a few studies that have dealt with the course and possible treatment options of IgLON5 antibody encephalopathy. In this study the clinical case of a female patient with IgLON5 antibody disease and the response to treatment is described. Here we report on the case of a 67-year-old female patient who showed cognitive deterioration, gait difficulties, and chronic obstructive sleep disorder. The diagnostic course showed a positive anti-IgLON5 serum and anti-IgLON5 IgG antibodies in cerebrospinal fluid. The patient was subsequently treated with high dosage i.v. methylprednisolone, i.v. immunoglobulins and plasmapheresis. Neuropsychological tests showed cognitive deficits in different domains, including verbal and visual memory. Both, neuropsychological deficits and antibody titer, showed an improvement after plasmapheresis. The presented case shows that IgLON5 disease can present with rapidly progressing cognitive deterioration as the prominent symptom, adding to the variety of clinical signs in this disorder. Testing for IgLON5-antibodies should be considered in patients with progressing cognitive decline, especially if accompanied by sleep disorders or neurological symptoms like oculomotor abnormalities, dysautonomia or bulbar signs.

## Introduction

IgLON5 antibody-associated encephalopathy is a rare autoimmune central nervous system disorder which also can present with signs of a tauopathy. Clinically, it is mainly characterized by sleep or sleep breathing disorder, and symptoms of a bulbar dysfunction, movement disorders and gait abnormalities (1, 2). The clinical spectrum of this disorder is still broadening, since the number of reported cases is still low, and cognitive decline as a symptom has been described in 40% of patients, even causing dementia (3). An early treatment with immunosuppressant or immunomodulatory drugs seems to be effective, but even with therapy the greater part of so far described patients suffered from chronic progression or remaining deficits (4, 5). Regarding its low prevalence in the general population, only a few articles have addressed symptoms and the course of the anti-IgLON5 disease. Consequently, the pathogenesis, the treatment, and, above all, the precise function of the IgLON5 antibody almost remain unclear (1–3). Here, we present a patient suffering from anti-IgLON5 disease with cognitive deficits in different domains, including verbal and visual memory, verbal fluency, and visuoconstructive performance, highlighting the variety of cognitive signs that can occur in this disorder.

## Patient and Methods

A 68-year old woman was introduced to the department of neurology in January 2018 with cognitive impairment since August 2017 and decreasing independence in everyday life. Moreover, the patient reported severe sleep problems. Polysomnography revealed chronic obstructive sleep disorder with recommendation for CPAP treatment and daytime sleepiness. In 2013, a gastric bypass surgery was conducted. In 2016, the woman suffered a minor stroke in the territory of the left middle cerebral artery with consecutive reduced fine motor skills of the right hand. Additional diseases included pulmonary disorder, depressive syndrome, and diabetes mellitus type 1 which was accompanied by diabetic polyneuropathy and diabetic retinopathy. Apart from the cognitive decline, the neurological examination revealed gait problems with small steps and ataxia, and missing tendon reflexes of the lower limbs, which was so far ascribed as signs of diabetic neuropathy.

During the course of the disease, gait difficulty increased and falling occurred frequently.

The diagnostic work-up showed a positive anti-IgLON5 serum (1:1,000), whereas no additional anti-neuronal antibody was detected [tested antibodies: Hu, Yo, Ri, CV2, amphiphysin, Ma2/Ta, Zic4, GAD65, Tr (DNER), Recoverin, Sox1; EUROLINE Blot; NMDA-R, AMPA-R, GABA-B, LGI-1, CASPR2, DPPX, glycine receptors, mGluR1, mGluR5, GABA-A; Western Blot, Euroimmun AG, Lübeck, Germany]. The MRI of the neurocranium was unobtrusive without signs of encephalitis or neurodegenerative disorder. An EEG recording was inconspicuous as well. An examination of cerebrospinal fluid in February 2018 revealed anti-IgLON5 IgG antibodies (1:3.2) with a normal cell count, a normal protein level, and negative oligoclonal bands.

## Therapeutic Interventions

**Figure 1:**
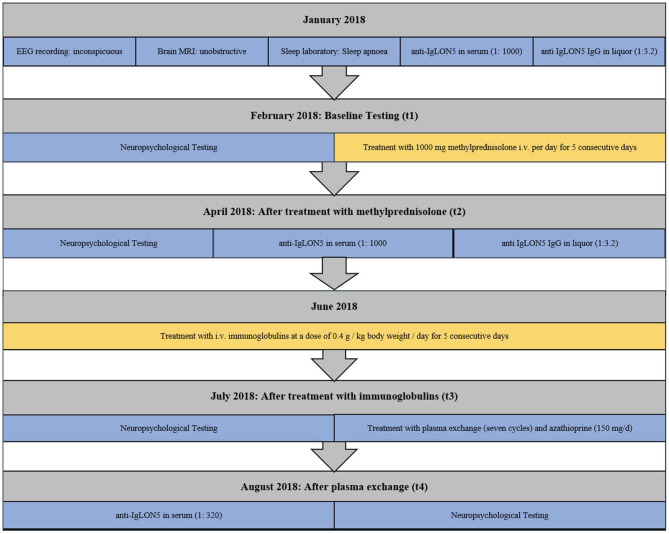
Timeline displaying the evolution of our case: in the gray boxes, the date has been reported; in the blue boxes is a list of the investigations that have been performed; and in the yellow boxes, we have displayed the therapies.

The patient was initially treated with 1,000 mg of methylprednisolone i.v. per day for five consecutive days. After the methylprednisolone treatment, cognitive testing was performed in April 2018 (t2). The laboratory follow-up showed a persistent high antibody titer in the serum (1/1,000) and a positive cerebro-spinal fluid (CSF) titer (1/1). Because a second treatment with methylprednisolone could not improve the objectifiable deficits, an escalation of immunomodulatory therapy was required. In June 2018, the patient was treated with i.v. immunoglobulins (Privigen) at a dose of 0.4 g/kg body weight/day for five consecutive days, but still, a progressive worsening of the cognitive state with progressive impairment in the daily life activities had to be reported. Cognitive testing was completed again after immunoglobulin therapy in July 2018 (t3), verifying the cognitive decline. This required the further escalation of the therapy during the following weeks, including seven cycles of plasmapheresis in August 2018 and long-term oral therapy with azathioprine (150 mg/d). Thereafter, neuropsychological testing showed improved results (t4), which for the first time were also reflected by decreasing serum antibody titres (from 1/1,000 to 1/320). Figure 1 illustrates the process of diagnosis and therapy.

## Diagnostic Assessment

For the assessment of cognitive functioning, the German version of the neuropsychological battery of CERAD-PLUS (6, 7) was administered. This test battery provides a profile of cognitive impairment based on demographically (age, education, gender) adjusted z-scores. Subtests of the CERAD-PLUS include verbal (animal category) and phonological word fluency (S-words), the short (15-item) version of the Boston Naming Test (object naming), verbal memory (word list learning, word list recall, word list discriminability, and a 10-word list memorization), visuoconstructive performance (copy of four geometric figures), and visual memory (free recall and constructional practice saving). Saving scores are defined as the percentage of correctly recalled items when compared with the last learning trial, and discriminability is the percentage of hits vs. false alarms during word recognition. Selective attention and cognitive flexibility were assessed with Trail Making Tests A and B.

With the Memory Clinic Basel, a z-value below −1.3 (roughly equivalent to the 10% percentile) is evaluated as an indication of a conspicuous test performance. For single-case analyses of changes in cognitive functioning Jacobson's Reliable Change Index (RCI) was used. In this context, pre- and posttreatment scores were converted into z values (*M* = 0, *SD* = 1). The RCI allows one to draw a significant and reliable conclusion at scores of ±1.96. For reliability, measures of test-retest reliability were used.

$$\begin{array}{l} RCI\;=\;\frac{\left(pretreatment\;score-posttreatment\;score\right)}{SE_{diff}}\\ SE_{diff}=\sqrt{2xSEM^{2}};\;SEM=SD\;x\;\sqrt{\left(1-r\right)} \end{array}$$

## Outcomes and Follow-ups

Cognitive assessment before treatment in February 2018 revealed deficiencies in most CERAD-PLUS subscales. For verbal memory, visual memory and visuoconstructive performance results were below average. For selective attention assessed with Trail Making Test A, and for cognitive flexibility tested with Trail Making Test B, performance was also below average, and the same was the case for semantic-categorical verbal fluency and phonemic verbal fluency. Strikingly, with the Boston Naming Test, the results showed performance that was above average. With the Mini-Mental-State Test, the results were within one standard deviation around average. For raw data and z scores, see Table 1. After methylprednisolone treatment, the patient showed remarkable improvements in verbal memory compared with the pre-test. In terms of visual memory, she showed better performance, although deficits could still be noted in the “draw figures” test. Regarding selective attention, she still reached a result below average, although a trend toward improvement was found. The performance regarding cognitive flexibility and verbal fluency was improved compared with the pre-test and could be noted in the average range. With the Mini-Mental-State Test, the results were improved compared with the pre-test. Semantic-categorical verbal fluency showed a similar result to the previous test (see Table 1). The RCI revealed a significant positive change in cognitive flexibility (RCI = −2.62) and on the Mini-Mental State Test (RCI = −1.96) (see Table 2).

**Table 1:** CERAD-PLUS subscales and results in all categories with raw values and standardized z values.

	**Baseline**		**t2**		**t3**		**t4**	
			**Post methylprednisolone**		**Post immunoglobulins**		**Post plasmaphereses**	
			**treatment**		**treatment**		**treatment**	
**CERAD-PLUS**	**Raw value**	**z value**	**Raw value**	**z value**	**Raw value**	**z value**	**Raw value**	**z value**
Verbal fluency	12	−1.49	12	−1.49	10	−1.93	14	−1.07
Boston naming test	15	1.47	14	0.05	15	1.47	14	0.05
Mini-mental state	28	−0.9	30	1.28	24	−3.19	28	−0.9
Word list learning	16	−1.76	19	−0.82	14	−2.39	16	−1.76
Word list recall	3	−2.19	5	−1.25	1	−3.25	5	−1.25
Word list savings (%)	50	−2.26	72	−1.01	17	−3.69	84	−0.23
Word list recognition (%)	85	−2.19	95	−0.97	65	−3.77	90	−1.66
Draw figure	6	−2.67	7	−2.15	8	−1.6	8	−1.6
Figure recall	3	−2.14	6	−0.97	7	−0.54	8	−0.06
Figures saving (%)	50	−1.31	86	0.18	88	0.28	100	1.25
Trail making test A (sec)	102	−2.35	85	−1.91	75	−1.59	71	−1.44
Trail making test B (sec)	224	−1.71	135	−0.48	300	−2.28	192	−1.37
Phonological word fluency	5	−1.62	7	−1.03	4	−1.94	9	−0.5

*A z value below −1.3 is considered to be an indication of a test performance that deviates from the norm*.

**Table 2:** Reliability of CERAD-PLUS subscales and the result of RCI analyses regarding the changes between pre- and posttreatment performance.

		**Jacobson's Reliable Change Index (RCI)**					
	**Reliability Coefficient**	**Baseline vs. methyl-prednisolone**	**Baseline vs. immunoglobulins**	**Baseline vs. plasma-phereses**	**Methyl-prednisolone vs. immuno-globulins**	**Methyl-prednisolone vs. plasma-phereses**	**Immuno-globulins vs. plasma-phereses**
Verbal fluency	0.67	0	0.54	−0.52	0.54	−0.52	−1.06
Boston naming test	0.56	1.51	0	1.51	−1.51	0	1.51
Mini-mental state	0.38	−1.96^*^	2.06^*^	0	4.01^*^	1.96	−2.06^*^
Word list learning	0.62	−1.08	0.72	0	1.80	1.08	−0.72
Word list recall	0.64	−1.11	1.25	−1.11	2.36^*^	0	−2.36^*^
Word list recognition (%)	0.36	−1.08	1.40	−0.47	2.47^*^	0.61	−1.86
Draw figures	0,54	−0,54	−1.12	−1.12	−0.57	−0.57	0
Trail making test A (sec)	0.79	−0.68	−1.17	−1.40	−0.49	−0.73	−0.23
Trail making test B (sec)	0.89	−2.62^*^	1.22	−0.72	3.84^*^	1.90	−1.94

RCI values of ≥-1.96 indicate better test performance and values of ≥1.96 indicate worse test performance. In the range from −1.96 to +1.96, no significant change can be assumed.

For reliability for Verbal Fluency, Boston Naming Test, Mini-Mental State, Constructional Practice, Word List Learning, Word List Recall, and Word List Recognition see (8); for Trail Making Tests A and B see (9).

* *Marks significant differences*.

After treatment with immunoglobulins, the patient showed results below average in verbal fluency, verbal and visual memory, visuoconstructive performance, selective attention, and cognitive flexibility, as well as on the Mini-Mental State Test (see Table 1). Strikingly, the Boston Naming Test results showed average performance. The RCI revealed a significant negative change on the Mini-Mental State Test (RCI = 2.06) (see Table 2).

After treatment with plasmapheresis, the patient showed results below average on some subtests of verbal memory, selective attention, cognitive flexibility, and visuoconstructive performance (see Table 1). For verbal fluency, naming objects, the Mini-Mental State Test, and phonological fluency she now achieved average results.

Although the RCI did not show significant changes of pre- and post-treatment in any of the subtests, the patient showed the best results in verbal and phonological word fluency, selective attention and visual memory after plasmapheresis compared to baseline test and treatment with prednisolone and immunoglobulins. Performance also improved in verbal memory and cognitive flexibility compared to baseline test (see Table 2).

A statistical comparison of the therapy forms was also carried out (see Table 2). For treatment with immunoglobulins compared with treatment with methylprednisolone, the RCI revealed significantly worse performance on the Mini-Mental State Test (RCI = 4.01), in verbal memory “word list recall” (RCI = 2.36), in “word list recognition” (RCI = 2.47), and in cognitive flexibility (RCI = 3.84). No statistically significant differences were found for the comparison of methylprednisolone and plasmapheresis. For the comparison of immunoglobulins and plasmapheresis, the RCI showed significant improvements after plasmapheresis compared with treatment with immunoglobulins for “word list recall” (RCI = −2.36) and the Mini-Mental State Test (RCI = −2.06). Neither the sleep disorder nor gait difficulties improved with any therapy.

## Discussion

We here present a patient with IgLON5 disease with impaired verbal fluency, impaired verbal and visual memory, visuoconstructive deficits and reduced selective attention being the predominant neuropsychological symptoms. Only few cases of cognitive deficits in IgLON5 disease have been described so far (2, 3). The cases presented in the literature showed figural memory impairment, deficits in attention, executive dysfunction, as well as impaired verbal memory performance and even neuropsychiatric signs like hallucinations (10, 11). The patient reported here showed a combination of cognitive deficits in different domains, including verbal and visual memory, verbal fluency, selective attention and visuoconstructive performance, adding to the neuropsychological profile of IgLON5 disease. Verbal memory and fluency as well as visual memory showed a short-time improvement after treatment with high-dosage methylprednisolone. Interestingly, the patient showed the best performance in verbal and phonological word fluency, selective attention, visual memory, verbal memory and cognitive flexibility after plasmapheresis, accompanied by decreasing IgLON5 antibody-titer.

IgLON5-associated encephalopathy is a rare, but increasingly recognized disorder with a variety of clinical signs. Most commonly, sleep disturbances are reported together with neurological symptoms like gait difficulty, bulbar sings or movement disorders. Cognitive decline is present in up to 40% of patients and may represent the first symptom (3). Treatment options include immunosuppressants, such as intravenous steroids, plasmapheresis, and b-cell-depleting medication as rituximab. However, the treatment effects are not sharp, so often no clinical improvement can be achieved, and a progressive course or remaining deficits are common (4, 5). Bonello et al. reported an improvement in behavior and CSF parameters in a 45-year-old patient after treatment with prednisolone, immunoglobulins and plasmapheresis which led to a rapid improvement continuing over the next 2 years (12). Cagnin et al. reported a case in which treatment with intravenous steroids was not beneficial (13). Montagna et al. referred to a patient with IgLON5-associated disease who was treated with plasma exchange (14). This treatment led to cognitive improvement, and the anti-IgLON5 serum titer decreased from 1:10,000 to 1:320. This suggests that cognitive decline could be related to the titer of anti-IgLON5 antibodies and that the antibodies play a role in pathogenesis. This case is comparable to our patient, with cognitive deficits showing little improvement after corticosteroid or IVIg treatment, when after plasmaphereses a reduction of IgLON5-antibody titer and concurrent an improvement of the cognitive deficits, mainly verbal fluency, selective attention and visual memory, occurred (14). Neuropathological studies showed that in some patients with IgLON5-disease an accumulation of hyperphosphorylated tau-protein exists, predominantly in the hypothalamus and the brainstem, which might explain the sleep-associated symptoms (1, 15).

The patient presented here was tested four times with the CERAD-Plus test battery, for which unfortunately no parallel test versions are available. Although at least 2 months had passed between the tests, learning effects cannot be excluded. It should also be mentioned that no baseline tests were available, as the first test was done after symptoms occurred. However, the concurrent decline of antibody-titer together with improvement of the neuropsychological findings may be a hint for a relation between the antibody-titer and the clinical course of the disease, which needs to be objectified with greater patient numbers in the future.

Our case shows that a cognitive decline with impaired verbal and visual memory can be the leading symptom of an IgLON5 disease. The so far reported typical symptoms are sleep disorder and gait abnormalities or bulbar dysfunction, and cognitive symptoms show a broad variety from visuoconstructive dysfunction to hallucinations. This combination of symptoms is highly unspecific, making the diagnosis of IgLON5 disease based on the clinical symptoms difficult. The so-far described cases showed very different results after treatment, and the greater part of patients suffered a progression or remaining deficits. An early treatment in the course of the disease and timely escalation to plasmapheresis or B-cell-depleting antibodies seems to be recommendable.

## Concluding Remarks

IgLON5-associated encephalopathy is a comparatively new disorder with autoimmune and neurodegenerative properties that can manifest through various neurological and neuropsychological symptoms. We describe a patient with anti-IgLON5 disease with severe cognitive deficits as the main symptoms, with predominantly verbal and visual memory deficits, showing that a thorough diagnostic work up is necessary for this yet underdiagnosed disorder. Patients presenting with cognitive deterioration in combination with sleep disorder or sleep breathing difficulty and neurological symptoms as for example bulbar dysfunction, gait abnormalities, movement disorders like chorea or oculomotor abnormalities should be tested for the presence of IgLON5-antibodies. Since the clinical spectrum of this disorder is still expanding, and the cognitive impairment seems to respond to early treatment, testing for IgLON5 antibodies should be initiated generously.

## Data Availability Statement

The original contributions presented in the study are included in the article/supplementary material, further inquiries can be directed to the corresponding author/s.

## Ethics Statement

Written informed consent was obtained from the individual(s) for the publication of any potentially identifiable images or data included in this article. Written informed consent was obtained from the patients' daughter for the publication of any potentially identifiable images or data included in this article.

## Author Contributions

JE was involved in the patient case, collected the necessary data, and drafted and finalized the manuscript. MT was involved in the patient case, delivered the necessary data, and critically revised the manuscript for intellectual content. TG and MJ were involved in the patient case and critically revised the manuscript for intellectual content. All authors contributed to the article and approved the submitted version.

## Conflict of Interest

The authors declare that the research was conducted in the absence of any commercial or financial relationships that could be construed as a potential conflict of interest.
